# Construction of a virtual simulation teaching system for medical imaging education: a single-center experience

**DOI:** 10.3389/fmed.2026.1870420

**Published:** 2026-07-02

**Authors:** Rong Xu, Wenchong He, Xinyuan Zhang, Ke Xu, Jiang Li, Yingkun Guo, Yun Wang, Huayan Xu, Bo Huang

**Affiliations:** 1 Department of Radiology, Medical Imaging Center, West China Second Hospital of Sichuan University, Chengdu, Sichuan, China; 2 Key Laboratory of Children's Medicine, Obstetric and Gynecologic and Pediatric Diseases, and Birth Defects and Related Diseases of Women and Children, West China Second University Hospital, Sichuan University, Chengdu, Sichuan, China; 3 State Key Laboratory of Oral Diseases, National Center for Stomatology, National Clinical Research Center for Oral Diseases, West China Hospital of Stomatology, Sichuan University, Chengdu, Sichuan, China

**Keywords:** medical imaging education, practical capabilities, practical skill training, virtual simulation teaching, cardiovascular imaging

## Abstract

Virtual simulation constitutes a multidisciplinary field that integrates computer graphics, dynamic simulation modeling, human–computer interaction, and artificial intelligence. It has evolved from an auxiliary tool into a core teaching methodology, becoming indispensable to modern medical education. Through the establishment of a high-fidelity digital ecosystem, this technology achieves the accurate reproduction of real clinical scenarios. It provides a reliable support platform for evidence-based teaching and practical clinical competency training. Medical imaging education is a critical component of clinical training, and the integration of virtual simulation technology has emerged as a key strategic approach to optimizing clinical teaching outcomes, bridging the gap between theoretical knowledge and clinical practice. Furthermore, medical imaging education aims to cultivate competent clinical imaging professionals with comprehensive practical capabilities. This descriptive single-center study focuses on the development, implementation, and evaluation of a specialty-oriented virtual simulation teaching system for medical imaging technology. The effectiveness of the system was evaluated by comparing pre- and post-training outcomes among participants with three different levels of technical experience. The key components of an effective virtual simulation teaching system include clear identification of user needs, selection of appropriate technological approaches, sufficient granularity in system design, and implementation of engaging interaction modalities. For the training assessment of the magnetic resonance imaging (MRI) section, the comprehensive skills related to fetal scanning improved in all subjects (54.19 ± 18.03 vs. 66.62 ± 12.41, *p* < 0.001) after virtual simulation training. Among technicians with different experiences, residents (42.38 ± 15.41 vs. 59.95 ± 12.63) and junior technicians (63.4 ± 12.13 vs. 73.07 ± 8.34, all *p* < 0.05) showed the most significant improvement after retraining. In contrast, the improvement among senior technicians (72.50 ± 7.89 vs. 73.83 ± 7.41, *p* = 0.117) was not significant. These findings suggest the potential application of virtual simulation in fetal prenatal screening training.

## 1 Introduction

Medical simulation technology enables the creation of simulated patients and clinical scenarios for teaching and practicing without relying on real patients (1). It provides an effective environment for students to acquire and practice clinical skills (2). With the increasing demand for standardized basic clinical skills training, simulation technology has been widely applied in multiple disciplines, including internal medicine, surgery, nursing, and rehabilitation medicine (3, 4). Simulation-based medical education has undergone three major developmental stages (5). Standardized patients (SPs) have been widely used in clinical skills training and assessment since the 1960s. SPs allow high-fidelity clinical communication training to be conducted in a low-risk environment by simulating authentic clinical conditions and scenarios (6). They are widely applied in medical history-taking exercises and psychiatric interviews, effectively enhancing the communication skills and clinical confidence of medical students (7, 8). The second stage involves three-dimensional (3D) printing technology. It is characterized by the ability to create accurate physical objects from digital models (9). This technology has shifted medical education away from a heavy reliance on cadaver dissection and two-dimensional (2D) imaging toward more realistic and interactive approaches. It has significantly influenced both skill transfer and the academic performance of learners (10, 11). Although debates persist regarding the accuracy of anatomical structure identification, these models generally provide novice learners with a realistic hands-on practice experience. The third stage refers to immersive learning environments based on virtual simulation technology (12, 13). Virtual reality (VR), augmented reality (AR), and mixed reality (MR) technologies create highly interactive and immersive learning environments (14).

Currently, virtual simulation has become an indispensable component of modern medical education (15), evolving from an auxiliary teaching tool into a core instructional modality. These technologies enable the training of complex procedures such as oral, cardiac, and orthopedic surgeries with reduced risk (16–18). They are particularly effective in facilitating rapid skill acquisition among novice learners. According to recent systematic reviews, the success of virtual simulation depends on several factors: alignment with educational programs, in-depth integration with curriculum objectives, validated design efficacy, and due attention to learners’ acceptance and user experience (19). Medical imaging education constitutes a vital component of clinical training. The application and systematic development of virtual simulation technologies have emerged as a critical strategy for optimizing educational outcomes.

However, research and development of virtual simulation systems in medical imaging education remain inadequate. This study systematically synthesizes existing experiences in the construction of virtual simulation-based teaching systems. It aims to develop a highly efficient teaching system tailored to specific institutional contexts and educational requirements. The findings and experiences derived from this study provide guidance for the systematic implementation of virtual simulation technologies in medical imaging education and facilitate their broader adoption and in-depth application in medical practice.

## 2 Construction of a virtual simulation system

### 2.1 Essential considerations

**Figure 1:**
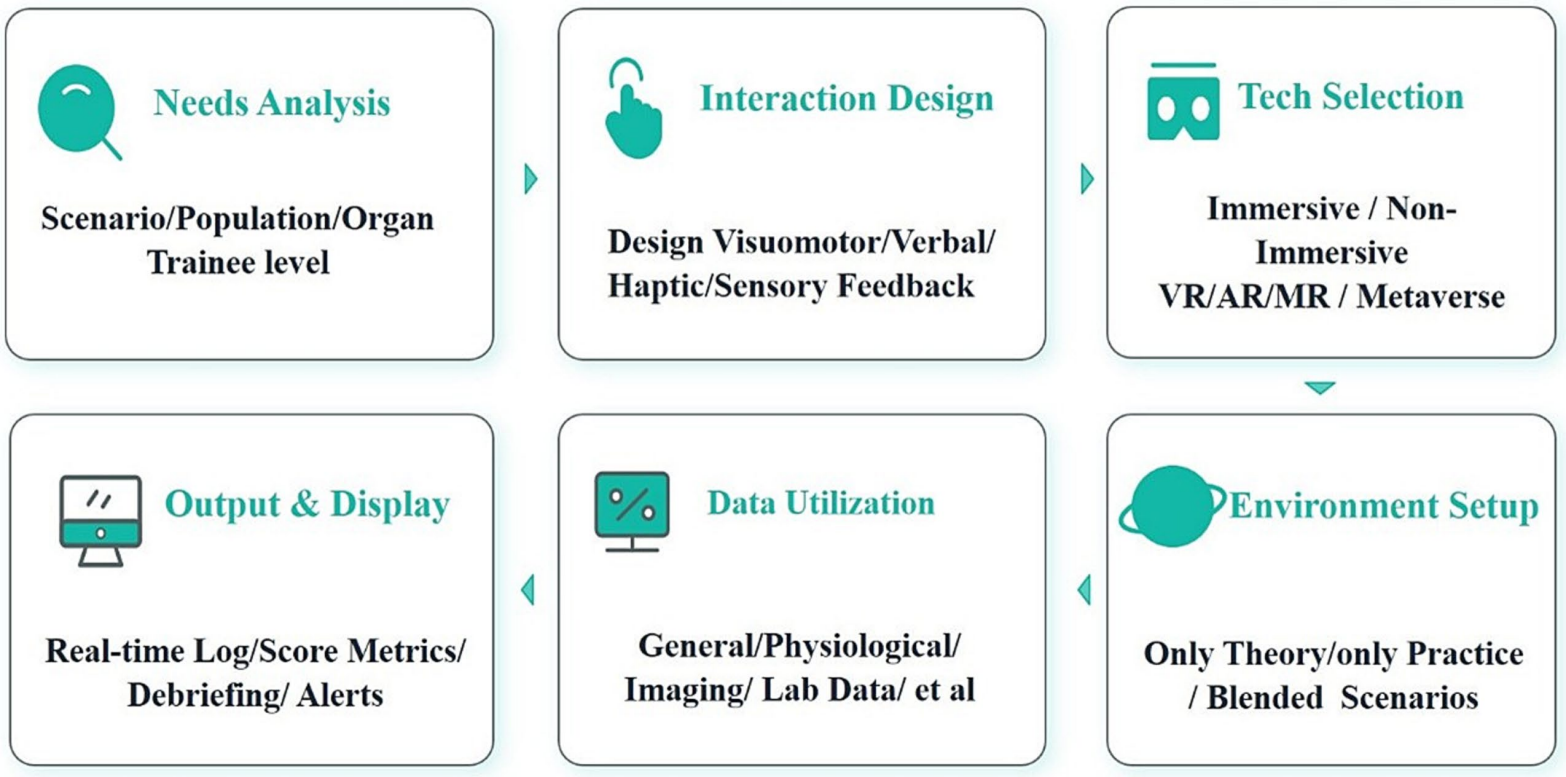
Design framework overview of virtual simulation system. There are six key dimensions to the design framework, including needs analysis, interaction design, technology selection, environment setup, data utilization, and output and display. VR: virtual reality; AR: augmented reality; MR: mixed reality.

Clinical practice integrates theoretical knowledge with practical skills. Therefore, the development of a virtual simulation system must be closely aligned with clinical needs and address the requirements of different end users. An effective system should incorporate six key dimensions (Figure 1), as outlined below:

(1) Needs analysis: A clear understanding of the target users is essential, as it forms the basis of an end-to-end needs assessment. The target user population and application scenarios should guide system development, with four specific aspects requiring careful consideration.

 (a) Clinical scenario appropriateness: Scenarios should reflect the most common and information-rich situations encountered in clinical practice.
 (b) Completeness of disease types: Selected diseases should cover the most common and representative clinical entities encountered in clinical settings, supported by a robust knowledge base and case repository spanning various disease categories.
 (c) Coverage of user groups: Different user groups possess varying levels of prior knowledge, clinical experience, and learning objectives.
 (d) Convenience of assessment and evaluation: Training and evaluation requirements vary across user groups at different stages of learning; thus, the focus of assessment should be tailored accordingly.

(2) Interaction design: This aspect defines how the system operates and is determined by the design strategy. Various interaction modalities are available. A user-centered design approach that benefits both service providers and end users is fundamental to developing an effective interactive interface.
(3) Technology selection: This dimension pertains to how the system is constructed, with multiple technical pathways available for implementation. Technolgy selection should be informed by prior case studies, aligned with the requirements of interaction design, and optimized with consideration of cost-effectiveness.
(4) Environment setup: This dimension addresses the context of system use, which is determined by specific scenario requirements. The choice of application scenario influences the presentation of the virtual environment and the associated technical considerations.
(5) Data utilization: Adequate granularity must be incorporated throughout system development. Opportunities for data collection and scenario enrichment should be embedded at each operational step, with key information appropriately annotated. This dimension is critical in determining the overall quality of the system.
(6) Output and display: As the final visualization interface, the system should include features for recording user operations and displaying training performance scores. Such functionalities are essential for facilitating post-training review and ongoing system optimization.

### 2.2 Construction of virtual training simulation system for fetal congenital heart disease (CHD) diagnosis

**Figure 2:**
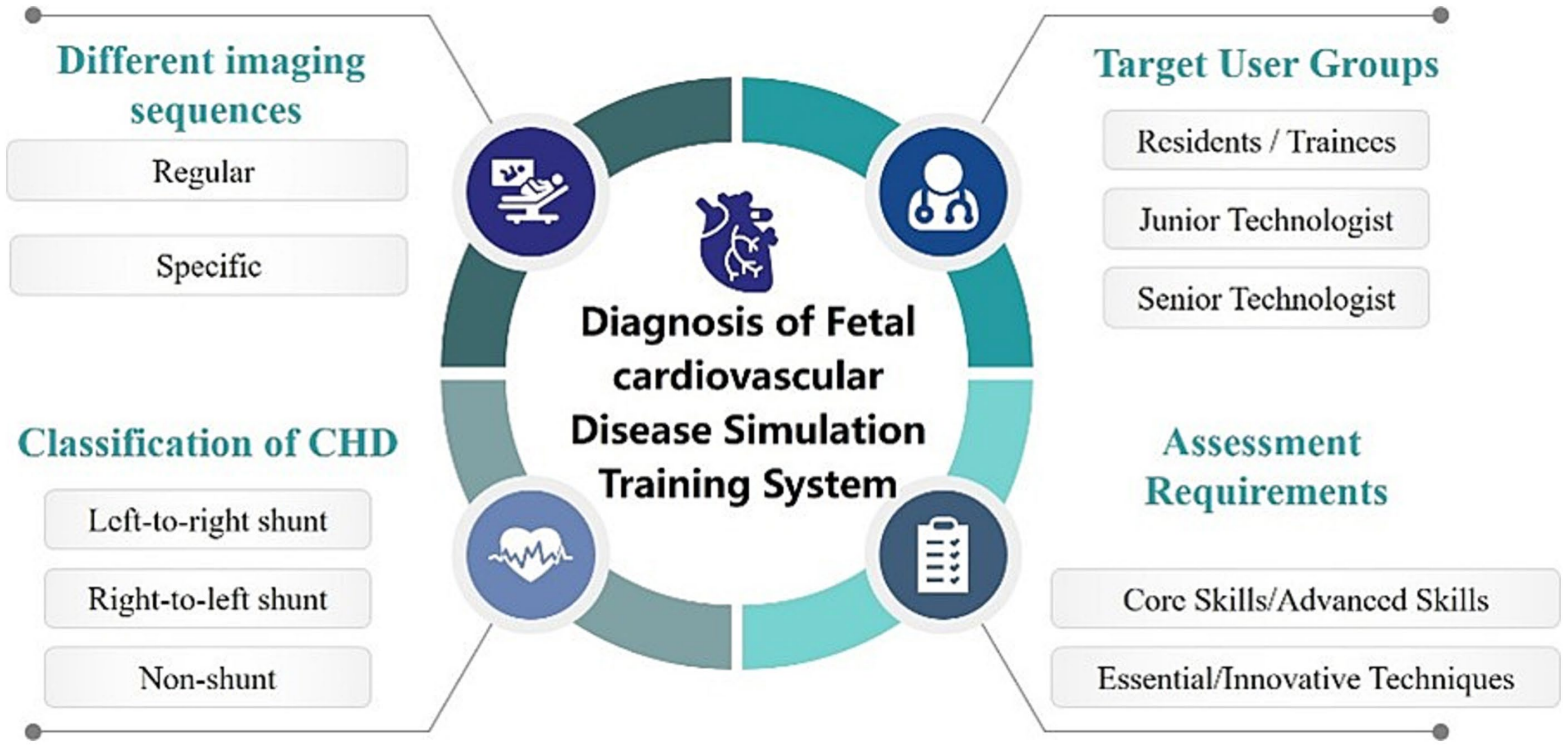
Needs analysis of the diagnosis of fetal cardiovascular disease simulation training system. Sort out the clinical needs and training key points of our center. CHD: congenital heart disease.

In teaching practice, we developed a virtual simulation system for training in fetal congenital heart disease imaging techniques (Figure 2).

#### 2.2.1 Needs analysis

Birth defects are a leading cause of infant mortality worldwide (20). Congenital heart disease (CHD) has the highest incidence among all birth defects, making it a key priority in birth defect prevention and clinical management (21). As the largest women’s and children’s hospital in Southwest China and a major referral center, our institution manages over 10,000 pregnant women annually, including numerous complex cases (22). Radiology specialists face numerous clinical challenges (23–25). In response, government initiatives have consistently emphasized the importance of standardized technical training and certification for birth defect prevention. These policies mandate that personnel involved in prenatal screening and diagnosis obtain appropriate qualifications. Additionally, they aim to strengthen capacity building among medical imaging professionals.

Magnetic resonance imaging (MRI) is a well-established clinical modality for CHD screening (26, 27). However, several challenges persist in clinical practice, including non-standardized examination workflows, inconsistent training in imaging techniques and diagnostic interpretation, lengthy training cycles for specialists, and persistently high rates of misdiagnosis and missed diagnoses (28, 29). To meet the diagnostic and screening needs of radiology practice, the target users of this virtual simulation system include all physicians and technologists involved in fetal diagnosis in clinical settings.

#### 2.2.2 Virtual environment setup

**Figure 3:**
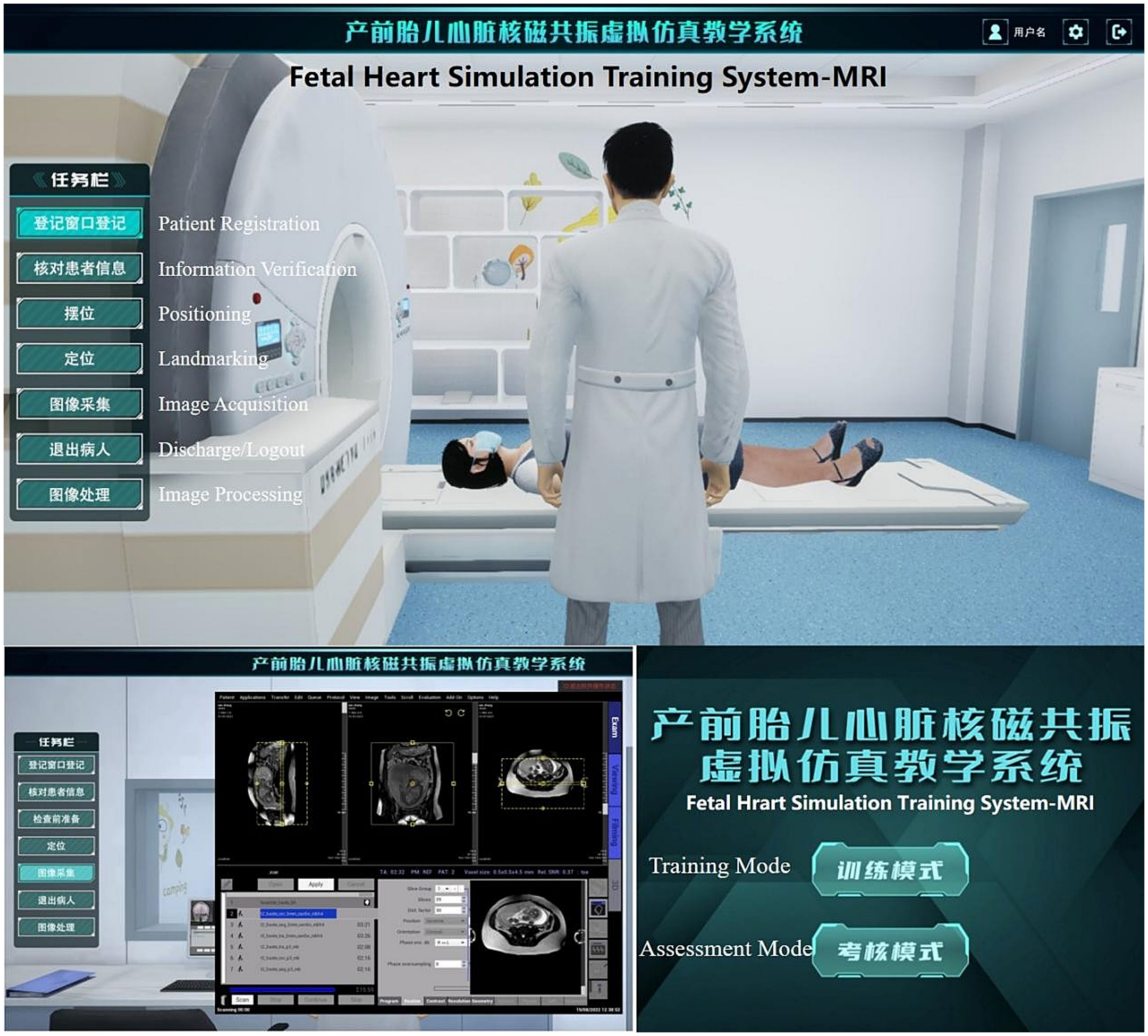
Virtual simulation system—MRI section. The system is structured according to the standard workflow for fetal MRI image acquisition.

Fetal cardiac MRI serves as a valuable complementary modality due to its multi-sequence and multi-parameter imaging capabilities (26). It is particularly recommended when visualization is suboptimal, including cases of maternal obesity, oligohydramnios, advanced gestational age, multiple gestations, or unfavorable fetal positioning. This system was designed to cover a full range of clinical scenarios by integrating a dedicated MRI module. This module covers patient positioning, sequence selection, parameter adjustment, and image acquisition (Figure 3). Special attention is given to addressing complex intra-scan scenarios, including variations in gestational age, fetal motion, maternal respiratory motion, and maternal obesity, along with appropriate parameter optimization strategies (29). The module demonstrates the impact of different parameter adjustments on image quality. All images were acquired from real-world clinical cases in our institution. Furthermore, tiered learning content is provided to accommodate the technical expertise and training requirements of radiologic technologists with varying levels of technical proficiency.

#### 2.2.3 Interaction design

The virtual teaching system provides a 1:1 simulation of real clinical scenarios and workflows. It structures the workflow chronologically from examination appointment, patient information verification and registration, patient positioning, imaging modality selection, to post-examination procedures (Figure 3). In the learning mode, operational guidelines for each step are presented via pop-up interfaces. In the training mode, a progressive “stage-by-stage” approach is used, which requires users to complete each step before proceeding to the next. Theoretical knowledge is seamlessly integrated into the virtual simulation system alongside authentic clinical cases. Learners are provided with repeated opportunities for hands-on practice, enabling skill proficiency to be developed through iterative training and assessment.

#### 2.2.4 Technology selection

The system was developed based on the mainstream Unity 3D engine (shown in the Supplementary materials). Built upon a virtual teaching framework, the system fully utilizes information technologies including human–computer interaction, augmented reality, and virtual reality. Students interact with the system using computer peripheral devices, including game controllers and mice, within a simulated hospital environment for MRI examinations.

#### 2.2.5 Data application

Based on hemodynamic characteristics, congenital heart diseases are categorized into three types: left-to-right shunt, right-to-left shunt, and non-shunt lesions (30). More than 10 different types of CHD have been incorporated into the system. The construction and application of cases both originated from the West China Second University Hospital of Sichuan University.

#### 2.2.6 Output and display

Residents undergoing standardized training, junior physicians, and senior physicians have distinct assessment requirements at different stages of professional development. The system accommodates these varying priorities by using tailored evaluation formats and ranking mechanisms for different user groups. For residents, assessment priorities focus on theoretical knowledge and recognition of simple disease types. For junior physicians, assessment requirements emphasize the complete examination workflow, image acquisition, and recognition of cardiac magnetic resonance imaging findings. For senior physicians, the evaluation criteria emphasize image acquisition and the identification of complex, uncommon pathological conditions using magnetic resonance imaging. After completion of training, the system generates a feedback survey addressing problems encountered during the training process. The software architecture, hardware requirements, development workflow, clinical data integration, system maintenance, scalability, and replication conditions are shown in the Supplementary materials.

### 2.3 Application assessment of the virtual training simulation system for fetal CHD

The application and evaluation of the MRI-based virtual simulation system were completed at a single center. This is a descriptive study based on a comparison of pre- and post-training assessments. The quantitative assessment data were obtained from a theoretical test.

**Figure 4:**
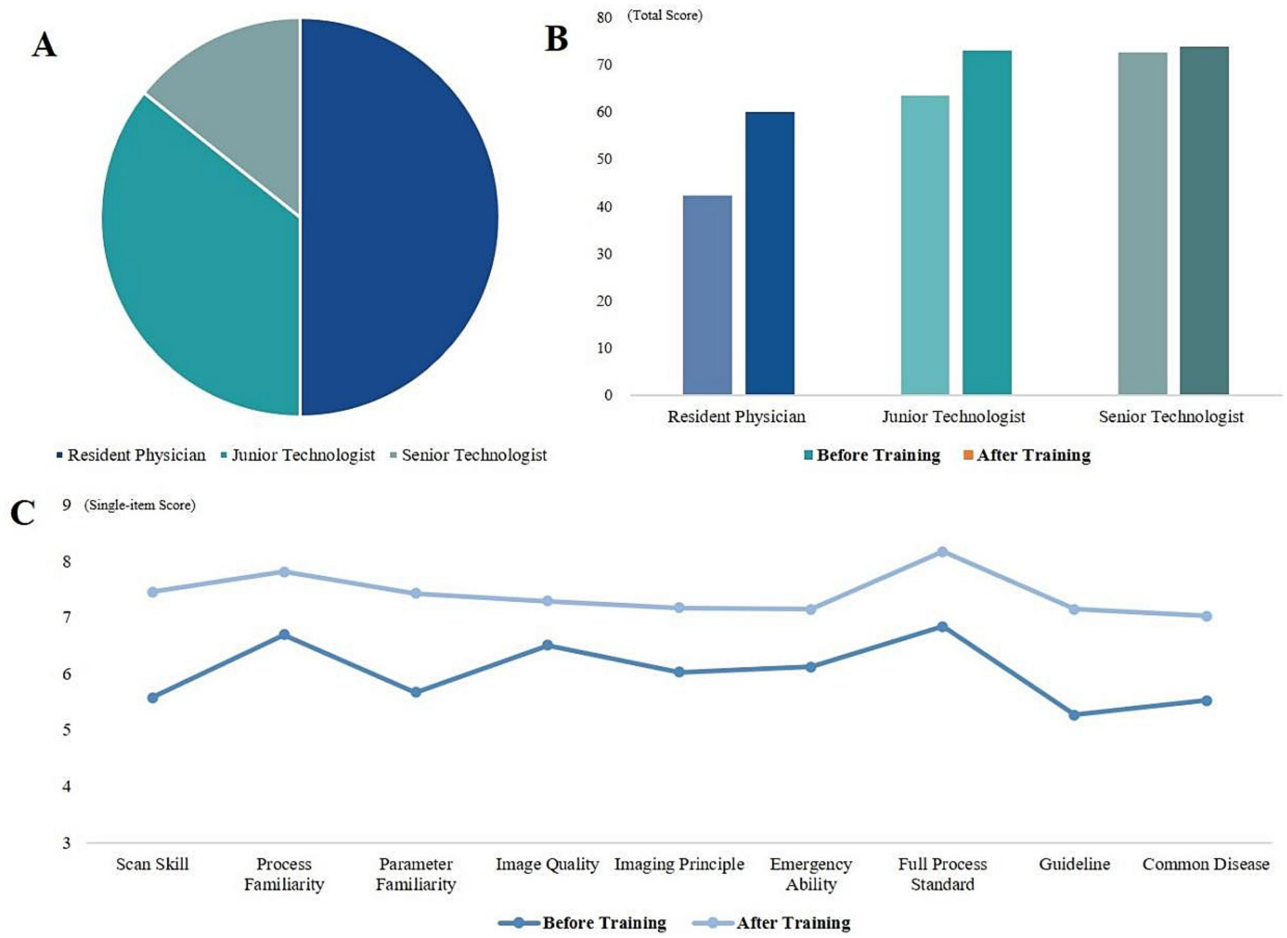
Virtual simulation system—training effect section. There were 42 technicians who underwent training in the fetal MRI technique. **(A)** The proportion of different subjects; **(B)** the total assessment of resident physicians, junior technologists, and senior technologists before and after the training; **(C)** in single-item evaluations of all subjects, the improvement levels before and after the training.

(1) Participant selection: For the MRI module, based on clinical needs and the grouping defined by the system (as shown in Figure 2: target user groups), participants were assigned to three groups according to their current level of experience: trainees, junior technologists, and senior technologists. This study was exempt from ethical review by the Ethics Committee of West China Second University Hospital because it did not involve the collection of private information from patients or participants and did not use invasive interventions. Training Implementation: The system was designed to accommodate the different needs of trainees with various levels of experience. Each group was directed to a different user interface during the training process. Details regarding the customized settings for each group are provided in the “Output and Display” section (Section 6) of the system description.
(2) Assessment design: The training content fully integrated current guidelines, expert consensus, and clinical experience. Key assessment points and corresponding test questions were developed collaboratively by one experienced fetal scanning technologist and one experienced fetal imaging diagnostician from our center, both of whom had substantial clinical and teaching expertise (>8 years of experience in fetal MRI). All assessments covered the following domains: scanning skills, process familiarity, parameter familiarity, image quality, imaging principles, emergency response ability, full-process standards, guidelines for fetal MRI, and fetal disease recognition. Each participant received two types of scores, both before and after training: domain-specific scores and a total score. Each domain was assigned a maximum of 10 points, with 5–10 questions per domain, and each question was assigned a different score (1–2 points). The total score was calculated as the sum of the scores obtained across all domains. To avoid the potential for unexplained score improvements that could arise from using identical assessment items, the specific test questions administered after the training differed from those used in the pre-training assessment. However, the complexity and core domains evaluated were kept consistent between the two assessments. The complexity of different questions before and after the assessment was determined by the question setter. All test items had a reference standard answer, which served as a benchmark for the evaluators (two experts) to measure participant performance by the same two experts. Both the pre- and post-training assessments were conducted by the same two experts to minimize bias arising from inter-rater variability. The same individuals who designed the assessment questions were also responsible for scoring the evaluations.
(3) Assessment process: The assessment process consisted of two steps. First, before training, all participants completed a theoretical test tailored to the three experience levels. The training period lasted 1 month. At the beginning of training, all participants received an introduction to the system and were granted access. Over the following month, no restrictions were placed on the frequency or duration of system use. All participants were blinded during both the pre- and post-training assessments. They were not informed that a formal evaluation would take place, nor were they aware of the specific content of the assessments.
(4) Statistical analysis: Due to inherent differences in baseline levels among the three groups, statistical analyses were performed independently for each experience level, with no multiple comparisons conducted across the groups. A paired-sample t-test was used to compare assessment scores before and after the training in the entire cohort and within the three groups. A *p*-value of < 0.05 was considered statistically significant.
(5) Results: There were 42 technicians who underwent training in MRI techniques and completed the assessment in a single center. Among all participants, resident physicians accounted for 50% (21/42), junior technologists for 36% (15/42), and senior technologists for 14% (6/42) (Figure 4A).

After virtual simulation training, the comprehensive skills of fetal scanning were improved (54.19 ± 18.03 vs. 66.62 ± 12.41, *p* < 0.001, Table 1).

**Table 1:** Assessment of comprehensive fetal scanning skills in all study participants before and after the training by a virtual simulation system.

Training assessment	Before the training (*n* = 42)	After the training (*n* = 42)	*p*-value
Total score	54.19 ± 18.03	66.62 ± 12.41	<0.001
Scan skill	5.57 ± 2.39	7.45 ± 1.40	<0.001
Process familiarity	6.69 ± 2.21	7.81 ± 1.37	<0.001
Parameter familiarity	5.67 ± 2.43	7.43 ± 1.64	<0.001
Image quality	6.50 ± 2.10	7.29 ± 1.64	0.001
Imaging principle	6.02 ± 2.24	7.17 ± 1.65	<0.001
Emergency ability	6.12 ± 2.00	7.14 ± 1.68	0.001
Full Process standard	6.83 ± 1.75	8.17 ± 1.43	<0.001
Guideline	5.26 ± 2.19	7.14 ± 1.77	<0.001
Fetal disease	5.52 ± 2.21	7.02 ± 1.72	<0.001

A *p*-value of <0.05 indicates statistical significance.

Among the technicians with different experience levels, the intermediate trainees (42.38 ± 15.41 vs. 59.95 ± 12.63, *p* < 0.05) and junior technicians (63.40 ± 12.13 vs. 73.07 ± 8.34, *p* < 0.05) showed the most significant improvement after retraining. In contrast, senior technicians did not exhibit a significant improvement [72.50 ± 7.89 vs. 73.83 ± 7.41, *p* = 0.117 (>0.05)] (Figure 4B). The degree of improvement before and after the training varied across individual evaluation items (Figure 4C). The pre-training and post-training item scores for technicians at different experience levels are presented in Table 2.

**Table 2:** Evaluation of comprehensive skills in fetal scanning among technicians with different levels of experience before and after retraining.

Training assessment	Resident physicians (*n* = 21)			Junior technologists (*n* = 15)			Senior technologists (*n* = 6)		
	Before the training	After the training	*p*-value	Before the training	After the training	*p*-value	Before the training	After the training	*p*-value
Total score	42.38± 15.41	59.95± 12.63	<0.001	63.4± 12.13	73.07± 8.34	<0.001	72.50± 7.89	73.83± 7.41	0.117
Scan skill	3.81 ± 1.83	6.81 ± 1.60	<0.001	7.07 ± 1.44	8.07 ± 0.80	0.006	8.00 ± 0.89	8.17 ± 0.75	0.771
Process familiarity	5.33 ± 2.20	7.14 ± 1.31	<0.001	7.80 ± 1.21	8.53 ± 0.99	0.010	8.67 ± 0.82	8.33 ± 1.37	0.530
Parameter familiarity	4.14 ± 0.20	6.71 ± 1.82	<0.001	6.93 ± 1.62	8.00 ± 1.07	0.002	7.83 ± 1.17	8.50 ± 1.05	0.102
Image quality	5.19 ± 1.97	6.52 ± 1.78	0.001	7.73 ± 1.44	8.13 ± 1.13	0.189	8.00 ± 0.63	7.83 ± 0.98	0.741
Imaging principle	4.71 ± 2.03	6.33 ± 1.68	<0.001	7.00 ± 1.65	8.00 ± 1.13	0.002	8.17 ± 1.17	8.00 ± 1.26	0.611
Emergency ability	5.00 ± 1.73	6.33 ± 1.65	0.012	6.93 ± 1.79	8.00 ± 1.36	0.002	8.00 ± 0.63	7.83 ± 1.17	0.695
Full process standard	5.76 ± 1.70	7.52 ± 1.54	<0.001	7.67 ± 1.05	8.00 ± 1.01	0.001	8.50 ± 0.55	8.83 ± 0.98	0.530
Guideline	4.05 ± 1.77	6.33 ± 1.91	<0.001	6.00 ± 1.81	7.80 ± 1.26	<0.001	7.67 ± 1.63	8.33 ± 1.03	0.102
Fetal disease	4.38 ± 2.18	6.24 ± 1.76	0.001	6.27 ± 1.58	7.73 ± 1.39	<0.001	7.67 ± 1.21	8.00 ± 1.10	0.465

A *p-*value of less than 0.05 indicates statistical significance.

## 3 Limitations

The effectiveness data of MRI technicians are currently available from a single center and a small cohort, and the content and implementation of the effectiveness evaluation were primarily based on the experience and judgment of senior technologists and physicians from a single center. The assessment may be subject to potential evaluator bias and lacked formal validation. Multicenter prospective controlled trials are warranted to provide more robust evidence (31). In addition, the current data only reflect theoretical and short-term training outcomes, which further limit the generalizability of the virtual simulation system for routine clinical implementation. Long-term theoretical and practical assessments are needed in future studies. Third, the current system integrates only core modules, which offers limited benefits for senior technicians. Ongoing updates will further expand its functionality to accommodate a broader range of training scenarios.

## 4 Conclusion

From a development perspective, clearly identifying user needs and selecting appropriate technological approaches are foundational. Furthermore, achieving high-quality virtual simulation depends on a fine-grained system design and engaging interaction modalities. The training of MRI technologists has yielded positive effectiveness. The impact is particularly significant for those at junior and intermediate experience levels. These findings suggest the potential application of virtual simulation in fetal prenatal screening training.

## Data Availability

The raw data supporting the conclusions of this article will be made available by the authors, without undue reservation.
