# Supplementary material for: Construction of a virtual simulation teaching system for medical imaging education: a single-center experience
Source: Front Med (Lausanne). 2026 Jul 2;13:1870420. doi: 10.3389/fmed.2026.1870420 (PMC13373652; doi:10.3389/fmed.2026.1870420)
Supplement: Supplementary file 1 [file Data_Sheet_1.PDF]

3D Unity Technical Documentation

This specification provides standardized explanations for the three core dimensions of project software architecture, hardware requirements, and development pipeline, fully matching the core features of the project: purely standalone offline operation, fully configurable content, and oriented toward medical teaching development.

一、Software architecture

This project uses a Unity engine-based monolithic single-player layered architecture, with no dependence on any network services, no integration of third-party cloud SDKs, and no external data exchange. All business logic and educational content are 100% closed-loop on the local device; it adopts the design concept of 'complete decoupling of logic and data,' allowing all educational content, process rules, and interaction parameters to be driven locally through configuration, enabling content iteration without modifying the code.

1. Core Layered Architecture

**Architecture hierarchy**	**Core Functions and Description**
Engine Base Layer	Built on the Unity LTS stable version engine, it provides core capabilities such as underlying 3D rendering, 2D UI rendering, physics collisions, input event management, resource lifecycle management, and program lifecycle scheduling. All engine capabilities run locally, with no cloud dependency, and natively support both Windows and macOS platforms.
Global Configuration Management Layer	The core central module of the project is responsible for the standardized parsing of local JSON configuration files, format validation, exception fault tolerance, and local hot loading; it uniformly manages the configurable content of all business modules, including theoretical knowledge point texts, scanning parameters, scanning step nodes, tool matching rules, correctness determination logic, resource path mapping, etc., achieving 'configuration-driven business,' allowing full teaching content adjustments without code modifications.
Resource Dynamic Management Layer	Linked with the configuration management module, responsible for on-demand dynamic loading, memory control, and release of local resources; supports precise reading of resources such as images, 3D models, teaching materials, videos, and audio in local folders, and

**Architecture hierarchy**	**Core Functions and Description**
	supports direct replacement and updating of local resources without needing to repackage the program
Business Function Module Layer	Corresponding to the four core teaching modules of the project, all modules are driven by configuration files, with low coupling between modules and the ability to be independently expanded. The functions are fully consistent across both platforms:<br><br>1. Theoretical Learning Module: A configurable chapter-based teaching module, where knowledge point texts, chapter structure, and illustration resources are all defined locally via JSON. It supports chapter navigation and local storage of learning progress.<br><br>2. Tool Cognition Module: A standardized tool cognition module, with sequence names, description texts, detailed illustration / 3D model resource paths, and interaction rules all locally configured, dynamically loaded and rendered. Adding or modifying sequences does not require code adjustments.<br><br>3. Scanning Simulation Module: The core interaction module, implementing a stepwise scanning process control based on a finite state machine; the node logic, trigger conditions, required tools, operation specifications, and correctness verification rules for before-during-after inspection processes are all fully defined via local JSON configuration. It has a built-in step verification engine that compares the user's tool selection and operational behavior in real time, automatically proceeding to the next node upon completing single-step verification, forming a fully local closed-loop workflow.
Local Data Persistence Layer	Based on Unity's native cross-platform local storage solution, responsible for locally storing users' learning progress, operation records, and module completion status. All data is retained only on the current device, with no data uploads or cloud synchronization.
Interactive Unified	Unified adaptation for mainstream input devices on both platforms: Windows adapts to mouse, keyboard, and touch screen; macOS adapts

Architecture hierarchy	Core Functions and Description
Management and Control Layer	to mouse / Magic Mouse, keyboard / Magic Keyboard, and multi-touch trackpad. Standardized management of the distribution and response of interaction events ensures consistency of cross-platform interaction experience across all modules.

2. Core features of the architecture

1. **1. Completely offline secure closed loop: no network requests, no external data interactions, no user privacy data uploads, fully meeting the data security and privacy protection requirements of medical teaching scenarios；**
2. **Fully configurable for all operations: Teaching content, process rules, and interaction parameters can be 100% configured through local JSON files, allowing the medical team to independently update content without developer involvement.；**
3. **Low coupling and high scalability: The layered architecture decouples underlying logic from business content, supporting the addition of new scanning processes, new sequences, and new teaching knowledge points without needing to refactor the core code.**
4. **Cross-platform high compatibility: Natively supports both Windows and macOS platforms. The program package comes with a complete runtime environment, requiring no additional dependencies or administrator privileges. It can be run immediately after extraction or dragging, making it suitable for batch deployment in teaching labs.**

二、Hardware requirements

This project supports native offline operation on both Windows 64-bit and macOS platforms, with fully identical functionality on both platforms. All content is configurable and operates entirely offline, with no network requests or external data interactions, and no mandatory dependence on dedicated peripherals. It can run fully on mainstream desktop devices and is suitable for various environments such as school computer labs and personal learning. Specific hardware requirements are divided into minimum operating configurations and recommended teaching configurations, covering adaptation needs for different devices.

1. Basic operating environment

Windows Platform

Target Platform: Windows 64-bit desktop operating system (compatible with all stable versions of Windows 10/11)

Operating Mode: Portable, no installation required, run immediately after extraction, no network adapter required (can run completely offline), no mandatory administrator privileges required

Peripheral Requirements: Standard mouse + keyboard sufficient for full operation, compatible with multi-touch screens, scanning simulation interaction peripherals, and other extended devices**macOS 平台**

- Target platform: macOS native universal binary application, natively supporting both Apple Silicon (M series) and Intel x86_64 dual-architecture Mac devices, no Rosetta 2 translation required, no compatibility loss
- Compatible system versions: Official stable versions of macOS 12 Monterey and above are the minimum compatible versions
- Operating mode: Green portable version, the App package can be run by simply dragging it into the 'Applications' folder, works normally in a completely offline environment, no network adapter or forced administrator privileges required
- Peripheral requirements: Standard mouse / Magic Mouse + keyboard / Magic Keyboard is sufficient for full operation, compatible with multi-touch trackpads, touch screens, scanning simulation interaction peripherals, and other extended devices.
- 2. Detailed configuration requirements

Configuration Type	Windows Minimum operating configuration	Windows Recommended Teaching Configuration	macOS Minimum operating configuration	macOS Recommended Teaching Configuration
Operating System	Windows 10 64	Windows 10/11 64	macOS 12 Monterey	macOS 14 Sonoma
Processor	Intel Core i5-6500 / AMD Ryzen 5 1500X	Intel Core i7-10700 / AMD Ryzen 7 5700X	Intel Core i5-7500 / Apple M1	Intel Core i7-10700 / Apple M2 Pro
RAM	8GB DDR4	16GB DDR4	8GB DDR4（Intel Mac）/ 8GB （Apple Silicon）	16GB DDR4（Intel Mac）/ 16GB（Apple Silicon）

Configuration Type	Windows Minimum operating configuration	Windows Recommended Teaching Configuration	macOS Minimum operating configuration	macOS Recommended Teaching Configuration
Graphics card	NVIDIA GeForce GTX 1050Ti / AMD RX 560 2GB+，DirectX 11	NVIDIA GeForce RTX 2060 / AMD RX 6600 6GB+，DirectX 12	Intel UHD Graphics 630 / AMD Radeon Pro 555 （Intel Mac）；Apple M1 GPU （Apple Silicon），Metal API	AMD Radeon Pro 5500M （Intel Mac）；Apple M2 Pro 16 GPU （Apple Silicon）， Metal API
Local Storage	Remaining available space≥10GB，SSD	Remaining available space≥20GB NVMe SSD	Remaining available space≥10GB SSD	Remaining available space≥20GB NVMe SSD

三、**Development Pipeline**

This project uses the Unity engine standardized cross-platform standalone software development pipeline, covering the entire process of 'requirement definition - content production - development integration - testing and verification - build and release.' The entire process can be completed offline in a closed loop, while also supporting parallel collaboration between the medical content team and the development team, significantly reducing content iteration costs, natively supporting synchronous build and release on both Windows and macOS platforms.。

1. Pre-requisite Requirements and Medical Content Production Stage

1. Standardization of requirements alignment: Deeply align with the West China medical expert team on the core knowledge points of scan teaching, clinical operation specifications, scanning process standards, and radiation protection requirements, clarifying the business boundaries, interaction rules, and medical content review standards of the four major modules.；
2. Structured content production: The medical team is responsible for producing standardized teaching content, including theoretical knowledge points, sequence parameters, 15-step scanning procedure specifications, criteria for determining correct

or incorrect operations, and radiation protection requirements, which are converted into configurable structured data.；

3. Digital Asset Production: Based on medical clinical standards, create fetal ultrasound/MRI scan 3D models, scan scene models, UI materials, educational illustrations, and other digital assets; establish standardized resource naming and path conventions; store them in local resource folders for configuration file mapping and usage; resources are fully compatible across both platforms.。

2. Development and Integration Phase

- **Core development toolchain**

• Core Engine: Unity 202x.x LTS Long-Term Support stable version, natively supports building on both Windows and macOS

• Code Development: VS Code / Rider integrated development environments, using C# language to develop cross-platform business logic

• Version Management: Git version control system to achieve full version management of code, configuration files, and digital assets, and support team collaboration

• Content Configuration Tools: Supports editing JSON configuration files with general text editors such as VS Code and Notepad, no professional development skills required, allowing the medical team to independently complete content configuration and updates

Core Development and Integration Log

1. Underlying Framework Development: Build a cross-platform layered architecture, develop common underlying modules such as a unified configuration parsing engine, dynamic resource loading system, scanning step state machine, interaction control, and local persistence, achieving complete decoupling of business logic and educational data, and adapting to underlying features of both platforms;

2. Business Module Development: Based on the underlying framework, develop common interactive logic, UI interfaces, process control, and judgment rules for the four major business modules. All variable content reserves configuration interfaces, with hardcoding only retaining general logic, achieving fully unified business logic across both platforms

3. Standardized Configuration System Construction: Define a JSON configuration structure and field specifications that cover all business operations, write configuration documentation, covering all configurable items including text content, resource paths,

scanning steps, tool parameters, and judgment rules, with configuration files being fully compatible across both platforms;
4. Content and Program Integration: After the medical team completes editing the configuration files and replacing resources, they can preview the effects directly in the Unity editor in real time without modifying the code, quickly completing the integration of educational content and the program without needing to adapt to platforms separately.
3. Testing and verification phase

1. Unit Testing: Conduct unit tests on core underlying modules such as configuration parsing, resource loading, and step determination to verify fault tolerance in scenarios like configuration format errors and missing resources, ensuring stable operation of core functions on both platforms;

2. Functional Integration Testing: Perform full-process testing on the two major modules, ultrasound and MRI, examining interaction logic, determination rules, and correctness of content loading in modules such as scan simulation, to ensure complete functional consistency across both platforms;

3. Medical Professional Verification: The center's team conducts a comprehensive professional review to validate the clinical accuracy of teaching knowledge points, scanning procedures, and operational norms, while synchronously optimizing content through configuration file adjustments, ensuring content updates take effect on both platforms simultaneously;

4. Compatibility Testing: Conduct offline operation tests on Windows devices with different configurations, teaching lab environments, as well as Mac devices with different chips and system versions, to verify operational stability, rendering effects, and compatibility across different hardware and system versions;

5. Deployment Scenario Testing: Perform specialized tests for scenarios such as bulk deployment in computer labs, operation without administrator privileges, and local configuration file updates to ensure the robustness and maintainability of programs on both platforms.
4. Build and Release Phase
1. Standardized cross-platform build pipeline: Based on Unity's native Build Pipeline and using the IL2CPP compilation mode, it can simultaneously build native executables for both Windows 64-bit and macOS platforms. During packaging, all dependent runtime environments, configuration files, and resource files are automatically

integrated, achieving green single-package delivery;
2. Deliverable output: The final deliverables are divided into two independent offline packages for Windows and macOS, each containing the executable file, standardized configuration folder, and local resource folder. No installation, network, or additional dependencies are required. Simply unzip / drag and drop to run, supporting batch copying and deployment in teaching labs;
3. Iteration and maintenance support: For subsequent teaching content updates, only modification of the local JSON configuration file and replacement of materials in the corresponding resource folder are needed, without rebuilding the program package. This allows full content updates for both platforms, significantly reducing subsequent maintenance costs.
